# Oxygenation of adipose tissue: A human perspective

**DOI:** 10.1111/apha.13298

**Published:** 2019-06-02

**Authors:** Ioannis G. Lempesis, Rens L. J. van Meijel, Konstantinos N. Manolopoulos, Gijs H. Goossens

**Affiliations:** ^1^ College of Medical and Dental Sciences, Institute of Metabolism and Systems Research (IMSR) University of Birmingham Birmingham UK; ^2^ Centre for Endocrinology, Diabetes and Metabolism Birmingham Health Partners Birmingham UK; ^3^ Department of Human Biology, NUTRIM School of Nutrition and Translational Research in Metabolism Maastricht University Medical Centre^+^ Maastricht the Netherlands

**Keywords:** adipose tissue, hypoxia, inflammation, metabolism, obesity, oxygen

## Abstract

Obesity is a complex disorder of excessive adiposity, and is associated with adverse health effects such as cardiometabolic complications, which are to a large extent attributable to dysfunctional white adipose tissue. Adipose tissue dysfunction is characterized by adipocyte hypertrophy, impaired adipokine secretion, a chronic low‐grade inflammatory status, hormonal resistance and altered metabolic responses, together contributing to insulin resistance and related chronic diseases. Adipose tissue hypoxia, defined as a relative oxygen deficit, in obesity has been proposed as a potential contributor to adipose tissue dysfunction, but studies in humans have yielded conflicting results. Here, we will review the role of adipose tissue oxygenation in the pathophysiology of obesity‐related complications, with a specific focus on human studies. We will provide an overview of the determinants of adipose tissue oxygenation, as well as the role of adipose tissue oxygenation in glucose homeostasis, lipid metabolism and inflammation. Finally, we will discuss the putative effects of physiological and experimental hypoxia on adipose tissue biology and whole‐body metabolism in humans. We conclude that several lines of evidence suggest that alteration of adipose tissue oxygenation may impact metabolic homeostasis, thereby providing a novel strategy to combat chronic metabolic diseases in obese humans.

## 1 INTRODUCTION

Obesity is defined as a body mass index (BMI) of 30 kg/m^2^ or above, and is characterized by excessive expansion of white adipose tissue (WAT) mass. The global trend in the prevalence of obesity represents a major public health problem, with more than 700 million children and adults affected worldwide.^1^, ^2^, ^3^ Obesity predisposes to multiple comorbidities, like insulin resistance and type 2 diabetes mellitus (T2DM), cardiovascular disease (CVD) and various types of cancer,^2^, ^4^, ^5^, ^6^, ^7^, ^8^ although 10%‐30% of the obese individuals will not be present with a pathological metabolic profile.^9^ Nevertheless, this phenotype, often referred to as metabolically healthy obesity,^9^, ^10^, ^11^, ^12^ carries an increased risk to develop CVD and T2DM later in life as compared to normal weight individuals.^13^, ^14^, ^15^, ^16^ This has led to the view that the pathophysiology of obesity and its complications is driven by WAT dysfunction rather than an increase in WAT mass only.^10^, ^17^, ^18^, ^19^

**Figure 1:**
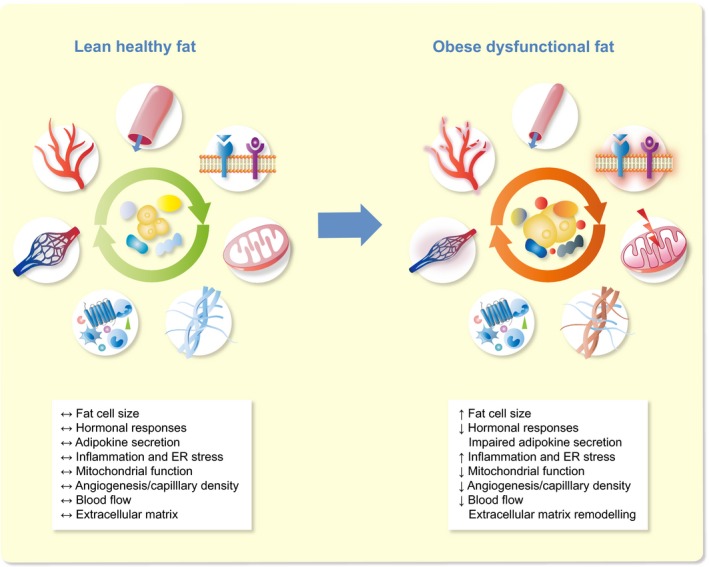
Characteristics of lean healthy and obese dysfunctional white adipose tissue. Adipose tissue dysfunction is characterized by adipocyte hypertrophy, impaired adipokine secretion, a chronic low‐grade inflammation, apoptosis, extracellular matrix remodelling, hormonal resistance, vascular rarefaction, decreased adipose tissue blood flow and altered metabolic responses, together contributing to insulin resistance and related chronic diseases. ER, endoplasmic reticulum

Dysfunctional WAT is characterized by adipocyte hypertrophy, impairments in lipid metabolism (including a reduced capacity to buffer the daily influx of dietary lipids, thereby contributing to ectopic fat accumulation), decreased adipose tissue blood flow and a state of chronic low‐grade inflammation (Figure 1).^18^, ^20^, ^21^ The presence of adipose tissue (AT) inflammation in obesity is well established, and several factors that contribute to the sequence of events leading to a pro‐inflammatory phenotype of obese AT have been identified, as extensively reviewed elsewhere.^10^, ^22^, ^23^, ^24^ Interestingly, more recent findings have provided evidence that the amount of oxygen in the adipose tissue microenvironment may also impact AT metabolism and inflammation, and WAT oxygenation may, therefore, be a key factor in the pathophysiology of AT dysfunction and related chronic diseases.^18^, ^25^, ^26^

In this review article, we will consider the role of WAT oxygenation in WAT dysfunction and its putative impact on the pathophysiology of obesity‐related metabolic and inflammatory diseases, with a focus on human studies. First, we will present a brief overview of the different aspects of WAT dysfunction in obesity. Thereafter, the oxygenation of WAT in obesity as well as the determinants of WAT oxygenation will be discussed. Next, the effects of WAT oxygenation on tissue (dys)function will be described, particularly in relation to inflammation and substrate metabolism. Finally, we will explore the effects of moderate hypoxia exposure on whole‐body physiology in humans.

## 2 ADIPOSE TISSUE DYSFUNCTION IN OBESITY

One of the main functions of WAT is the preservation of energy in the form of triacylglycerol (TAG) in response to a chronic‐positive energy balance.^27^ Adipose tissue has the capacity to expand at the cellular level by recruiting stem cells/pre‐adipocytes from the stroma‐vascular fraction (SVF) resulting in more adipocytes (hyperplasia), or by enlargement of existing adipocytes (hypertrophy).^28^, ^29^ However, it has been suggested that there is a set number of pre‐adipocytes that can be recruited, which seems to be genetically determined.^30^ Adipocytes can substantially increase in size but do have a certain expansion limit, implying that these cells have a maximum capacity of storing TAG.^23^, ^25^, ^31^, ^32^ What seems to be even more important than the maximal storage capacity is the ability to dynamically store lipids in the postprandial phase, the so‐called lipid buffering capacity, and to release fatty acids under fasting conditions.^33^ Hypertrophic WAT has been shown to have an impaired capacity to store meal‐derived fatty acids.^34^ As a consequence, more dietary lipids are diverted through the circulation to be stored in other tissues, which results in ectopic fat accumulation when lipid uptake exceeds lipid oxidation.^35^ The storage of excess lipids in non‐adipose tissues in obesity has important metabolic consequences, since this is closely associated with insulin resistance.^17^, ^23^, ^31^ Furthermore, hypertrophic adipocytes are characterized by a pro‐inflammatory phenotype, which may further aggravate insulin resistance.^24^, ^36^ Importantly, however, adipocyte inflammation also seems essential for healthy adipose tissue expansion and remodelling,^37^ suggesting that inflammation is not solely a pathological phenomenon. Noteworthy, medication used to treat type 2 diabetes may alleviate inflammation by reducing hyperglycaemia. However, the anti‐inflammatory effects of these agents are inconsistent, and it remains to be established whether their beneficial metabolic effects are mediated via modulation of chronic low‐grade inflammation.^38^

WAT inflammation is not only caused by secretion of pro‐inflammatory factors by adipocytes, but is also determined by infiltration of various populations of specialized, pro‐inflammatory immune cells 39, 40 such as macrophages.^27^, ^41^, ^42^, ^43^, ^44^ In rodents, macrophages can be divided into two major phenotypes, the pro‐inflammatory M1 and anti‐inflammatory M2 macrophages.^45^ M1 macrophages are activated by damage‐associated molecular patterns (DAMPs), cytokines such as IFN‐γ, and free fatty acids (FFA), acting as a major source of pro‐inflammatory cytokines, including tumour necrosis factor (TNF)‐α, interleukin (IL)‐1β, IL‐6, IL‐12 and IL‐23.^44^, ^46^, ^47^, ^48^, ^49^ In contrast, M2 macrophages play a role in tissue remodelling, and it seems that the M1/M2 ratio in WAT is critical in the pathophysiology of obesity, since M2 macrophages act as regulators and suppressors of inflammation, counterbalancing the pro‐inflammatory effects of M1 macrophages.^23^, ^50^, ^51^, ^52^, ^53^ Noteworthy, the macrophage phenotypes seem more complex, especially in humans where no clear division in M1/M2 macrophages is apparent.^54^, ^55^

In obesity, changes occur not only in the inflammatory cell population, but also in the extracellular matrix (ECM) of adipose tissue. The ECM consists of collagens, glycoproteins and proteoglycans, providing mechanical support and protection.^27^, ^56^ At the same time, the ECM interacts directly with the adipocytes’ signalling pathways in a dynamic way, affecting differentiation and expansion of the tissue.^22^, ^57^ The latter requires remodelling and alterations in the ECM composition, which has been associated with fibrosis and adipose tissue dysfunction in individuals with insulin resistance.^57^, ^58^

More recently, evidence has emerged that the oxygenation of WAT is altered in obesity, which may impact several aspects of WAT function and whole‐body physiology.

## 3 ALTERED ADIPOSE TISSUE OXYGEN PARTIAL PRESSURE IN OBESITY

Since alterations in the oxygenation of WAT may contribute to WAT dysfunction, as will be discussed later in this review, adipose tissue oxygen partial pressure (AT pO_2_) has been assessed in both rodents and humans. In addition to direct measurements of pO_2_, indirect methods to estimate WAT oxygenation have been applied (Table 1). The direct studies on WAT oxygenation have yielded conflicting findings, which are summarized in Table 2..^25^, ^34^, ^59^, ^60^, ^61^, ^62^, ^63^, ^64^, ^65^, ^66^

**Table 1:** Direct methods and surrogate markers used to determine adipose tissue oxygenation

Methods applied to assess adipose tissue oxygenation
*Direct*
Silastic tonometer^69^, ^70^, ^71^, ^72^
Polarographic micro clark‐type electrode^60^
Optochemical, continuous monitoring via microdialysis^59^, ^73^, ^74^, ^116^
Combined oxygen and temperature probe^57^
Needle‐type fibre‐optic oxygen sensor (rodents)^64^, ^65^, ^85^
*Indirect*
Arterio‐venous difference technique^34^
Gene expression of hypoxia‐responsive genes/proteins^63^
Pimonidazole hydrochloride^63^, ^66^

**Table 2:** Summary of studies in which adipose tissue oxygenation has been directly measured in humans

Study	Site of sWAT	Technique used	Participants' characteristics	AT pO_2_ (mmHg)
Kabon et al^69^ ^a^	Upper arm	Silastic tonometer	*Non‐obese*: n = 23 (12 M, 11 F); Age: 44 ± 9 y; BMI: 24 ± 4 kg/m^2^	Right arm: 54 (47, 64)^b^
				Left arm/wound: 62 (49, 68)^b^
			*Obese*: n = 23 (3 M, 20 F); Age: 44 ± 13 y; BMI: 51 ± 15 kg/m^2^	Right arm: 43 (37, 54)^b^
				Left arm/wound: 42 (36, 60)^b^
Fleischmann et al^70^	Upper arm	Silastic tonometer	*Non‐obese*: n = 15 (10 M, 5 F); Age: 43 y (13) c; BMI: 24 (3) kg/m^2^ c	57 (15)^c^
			*Morbidly obese*: n = 20 (4 M, 16 F); Age: 40 y (11) c; BMI: 46 (7) kg/m^2^ c	41 (10)^c^
Hiltebrand et al^71^	Upper arm	Silastic tonometer	*Lean*: n = 7 (2 M, 5 F); Age: 31 ± 6 y; BMI: 22 ± 2 kg/m^2^	52 ± 10
			*Obese*: n = 7 F; Age: 37 ± 6 y; BMI: 46 ± 4 kg/m^2^	58 ± 8
Pasarica et al^60^	Abdominal	Polarographic micro clark‐type electrode	*Lean*: n = 9 (5 M, 4 F); Age: 22.6 ± 3.3 y; BMI: 22.1 ± 1.0 kg/m^2^	55.4 ± 9.1
			*Overweight/obese*: n = 12 (6 M, 6F); Age: 38.9 ± 15.8 y; BMI: 31.7 ± 1.9 kg/m^2^	46.8 ± 10.6
Goossens et al^59^	Abdominal	Optochemical, measurement system	*Lean*: n = 10 M; Age: 55.8 ± 4.1 y; BMI: 23.4 ± 0.3 kg/m^2^	44.7 ± 5.8
			*Obese*: n = 10 M; Age: 59.6 ± 3.1 y; BMI: 34.2 ± 1.3 kg/m^2^	67.4 ± 3.7
Lawler et al^57^	Abdominal	combined oxygen and temperature probe	*Obese insulin sensitive*: n = 6 (4F/2M); Age: 36 ± 4; BMI: 32 ± 1 kg/m^2^	41.1 ± 1.2
			*Obese insulin resistant*: n = 6 (6F); Age: 37 ± 3; BMI: 34 ± 2 kg/m^2^	37.7 ± 2.4
			*Both obese groups*: n = 12 (10F/2M)	39.3 ± 1.5
			*Lean*: n = 4 (3F/1M); Age: 31 ± 3 y; BMI: 23 ± 1 kg/m^2^	53 ± 1.9
Kaiser et al^72^	Right upper arm	Silastic tonometer	*Morbidly obese*: n = 7; Age: 51 (35‐55); BMI: 67 (57‐71) kg/m^2^	Baseline (kPa): 6.8 (6.2‐7.6 [4.4])
			*Non‐obese:* n = 7; Age: 62 (53‐67); BMI: 26.5 (26‐29) kg/m^2^	Baseline (kPa): 6.5 (6.1‐7.5 [3.0])
Vink et al^74^	Abdominal	Optochemical, continuous monitoring via microdialysis	*Obese/overweight:* n = 15 (9F/6M); Age: 50.9 ± 2.1 y; BMI: Baseline: 31.1 ± 0.6 kg/m^2^	Baseline: 51.0 ± 1.6
			End of WS: 27.9 ± 0.5 kg/m^2^	End of WS: 41.3 ± 3.1
Goossens et al^73^	Abdominal	Optochemical, continuous monitoring via microdialysis	*Lean insulin sensitive*: men n = 7; Age: 58.6 ± 2.6 y; BMI 23.0 ± 0.3 kg/m^2^	40.4 ± 6.6
			*Obese insulin sensitive:* men n = 7; Age: 55.6 ± 2.8; BMI: 31.7 ± 0.8 kg/m^2^	56.1 ± 3.2
			*Obese insulin resistant*: men n = 7; Age: 56.9 ± 4.0; BMI: 33.1 ± 1.3 kg/m^2^	68.5 ± 4.4
			*Obese insulin sensitive:* women n = 7; Age: 50.6 ± 3.0; BMI: 30.5 ± 0.8 kg/m^2^	50.8 ± 2.5
			*Obese insulin resistant*: women n = 7; Age: 51.0 ± 2.3; BMI: 32.9 ± 1.8 kg/m^2^	62.3 ± 5.3
Vogel et al^116^	Abdominal & Femoral	Optochemical, continuous monitoring via microdialysis	*Obese/overweight:* n = 8 (F); Age:52.5 ± 1.8 y; BMI 34.4 ± 1.6 kg/m^2^	Abdominal: 62.7 ± 6.6
				Femoral: 50.0 ± 4.5

Abbreviations: AT, adipose tissue; BMI, body mass index; pO_2_, oxygen partial pressure (mmHg, if not indicated otherwise); kPa: kilopascal; sWAT, subcutaneous white adipose tissue; WS, weight stable period after diet‐induced weight loss.

a Measurements were taken on the morning the day after surgery.

b Median with 25th‐75th percentile.

c Results presented as means (SDs).

The presence of hypoxia in obese adipose tissue was originally shown in murine models of obesity.^18^, ^25^ Direct measurements of pO_2_ using needle‐type O_2_ electrodes showed that WAT oxygenation is lower in *ob/ob*, KKAy and diet‐induced obese mice as compared to lean controls.^18^, ^63^, ^64^, ^65^, ^66^, ^67^ In line, gene expression of several hypoxia‐related genes, including hypoxia‐inducible factor‐1 alpha (HIF‐1α), were also increased. Moreover, using pimonidazole hydrochloride, which stains hypoxic areas, it has been demonstrated that hypoxic areas were more prevalent in WAT of obese rodents.^18^, ^63^, ^64^, ^65^, ^66^, ^67^ However, it is worth mentioning that these rodent models of obesity are characterized by a rapid and massive gain in adipose tissue mass because of genotype and/or the diet that these animals received, which is not comparable to the more gradual development of obesity in most humans.^63^, ^64^, ^65^, ^66^, ^68^

So far, not many human studies examining WAT pO_2_ have been performed, and the results on WAT oxygenation are somewhat contradictory.^18^, ^59^ The first direct measurements of WAT pO_2_ in humans were made in individuals undergoing surgery.^69^, ^70^ It was found that morbidly obese individuals had lower pO_2_ levels in subcutaneous WAT (sWAT) of the upper arm as compared to lean subjects, determined the morning after surgery.^69^, ^70^ However, other studies in which sWAT oxygenation has been measured both during and after surgery showed opposite results, with increased or no significant difference in WAT pO_2_ between obese and lean individuals.^71^, ^72^ Notably, these initial studies assessed oxygenation in WAT of the upper arm, which is not of crucial importance for whole‐body metabolism. Moreover, the O_2_ levels measured in these studies could have been affected by the applied anaesthesia, and other factors related to morbid obesity.

Pasarica and colleagues 60 were the first to measure abdominal sWAT pO_2_ in humans, using a polarographic micro Clark‐type electrode. Overweight and obese participants, including patients with T2DM, had a lower AT pO_2_ compared to lean controls, which is in line with findings in rodents.^25^ Furthermore, it has been found that abdominal sWAT pO_2_ was higher in obese insulin sensitive and obese insulin resistant as compared to lean subjects, with no significant differences between the obese groups.^57^ Noteworthy, only four lean individuals were included in the latter study.

The presence of hypoxia in sWAT in obesity has been challenged by recent studies in humans. We have demonstrated a higher rather than lower pO_2_ in obese subjects with impaired glucose metabolism as compared to lean healthy, age‐matched individuals, despite lower adipose tissue blood flow (oxygen supply) in obesity.^59^ These findings of higher abdominal sWAT pO_2 _in obesity have been confirmed by very recent studies.^73^, ^74^ Abdominal sWAT pO_2_ was found to be higher in obese insulin resistant as compared to lean and obese insulin‐sensitive men, with no significant differences in WAT oxygenation between obese insulin‐sensitive and lean insulin‐sensitive men.^73^ Furthermore, this study demonstrated that AT oxygenation was positively associated with insulin resistance, even after adjustment for age, sex and body fat percentage, suggesting that AT pO_2_ may be more closely related to insulin sensitivity than obesity per se.^73^ To date, only one study investigated the effects of weight loss on sWAT pO_2_ in humans. In this study, overweight and obese individuals underwent a dietary intervention, consisting of a 5‐week very low calorie diet (VLCD, 500 kcal/d) and a subsequent 4‐week weight stable diet. It was found that VLCD‐induced weight loss markedly decreased abdominal sWAT pO_2_, which was paralleled by improved whole‐body insulin sensitivity.^74^

The striking differences in findings on sWAT pO_2_ between studies may be attributed to differences between study populations in terms of the onset and physical history (eg, weight cycling) of obesity and other subjects’ characteristics (eg, age, sex, ethnicity, presence of type 2 diabetes), the sWAT depot studied, and variation in the methodology used.^25^, ^59^, ^60^

In addition to direct measurements of sWAT pO_2_ in humans, several studies have used alternative approaches to indirectly estimate tissue oxygenation, including metabolic profiling of sWAT in vivo and the assessment of hypoxia‐responsive WAT gene expression. Hodson and co‐workers^34^ have measured metabolic fluxes across abdominal sWAT in vivo in lean, overweight and obese humans, and their findings strongly argue against any functional consequences of WAT hypoxia in obesity; in fact, the opposite might be true. More specifically, these authors demonstrated that the fasting lactate‐to‐pyruvate ratio, which is a potential metabolic signature of “hypoxia,” in arterial blood, was inversely correlated with adiposity. Using arteriovenous difference methodology with selective venous catheterization of abdominal sWAT, no significant association was found between WAT‐specific changes in lactate‐to‐pyruvate ratio and BMI. However, the proportion of glucose released as lactate and pyruvate in sWAT was strongly negatively correlated with BMI.^34^ Observational human studies examining hypoxia‐related genes as surrogate markers of WAT oxygenation have shown increased HIF‐1α expression in sWAT in humans with morbid obesity.^57^, ^75^, ^76^ Interestingly, HIF‐1α expression was higher in the SVF than in adipocytes, which might imply that the SVF is more sensitive to changes in oxygenation.^77^ Importantly, however, HIF‐1α mRNA expression seems not an appropriate marker for hypoxia.^78^ Also, upregulated genes in subcutaneous and visceral WAT of severely obese subjects that are under control of HIF were not responsive to hypoxia in adipocytes,^79^ which raises the question what pO_2_ threshold is required for activation of the HIF pathway in adipose tissue.^60^ Furthermore, genome‐wide association studies have shown a correlation between epigenetic methylation of the HIF3α gene in sWAT and BMI and WAT dysfunction markers.^80^, ^81^, ^82^, ^83^ Following bariatric surgery, there was a reduction in HIF‐1α mRNA expression in WAT.^84^ On the contrary, HIF‐1α gene expression was upregulated during weight loss induced by a low caloric diet.^74^

It is important to emphasize that a stronger mechanistic link exists between hypoxia and the spatial presence of HIF‐1α protein rather than its mRNA expression.^85^, ^86^ Further, HIF‐1α is not only regulated by oxygen levels, but also by growth factors including insulin.^87^ Therefore, metabolic disturbances such as insulin resistance and/or hyperglycaemia may also have marked effects on HIF‐1α protein stability,^87^ and may affect epigenetic modifications*.* This implies that one should be cautious when drawing conclusions about WAT oxygenation based on gene expression of classical hypoxia‐responsive genes such as HIF‐1α, GLUT1 and VEGF.^25^

Taken together, recent cross‐sectional and intervention studies that we have performed in our laboratory demonstrate higher rather than lower WAT pO_2_ in obese insulin resistant individuals, but findings on sWAT oxygenation (markers) in humans with obesity are conflicting. Thus, further investigation of determinants of sWAT oxygenation may help to better understand these discrepant findings.

### 3.1 Determinants of adipose tissue oxygenation in humans

WAT pO_2_ is the result of a delicate balance between O_2_ supply and consumption, which both seem to be altered in obesity. More specifically, differences in angiogenesis, capillary density and vascular function, together determining adipose tissue blood flow (ATBF), and the cellular demands affecting O_2_ consumption contribute to changes in WAT pO_2_.^18^, ^25^, ^68^

#### 3.1.1 Adipose tissue oxygen supply

Both structural (ie, capillary density) and functional (ie, vascular tone) aspects of the vasculature determine ATBF and, therefore, oxygen supply to WAT. There is substantial evidence that there is insufficient angiogenesis in WAT depots in obesity. Obese individuals show decreased adipose tissue mRNA expression of VEGF, the master regulator of angiogenesis and a HIF‐1α target protein.^59^, ^60^, ^88^ Pasarica and colleagues^60^ showed that capillary density was lower in overweight/obese humans, and found a positive correlation between VEGF expression and capillary density. The lower capillary density in WAT of obese individuals has been confirmed by our laboratory.^59^ Furthermore, it has been shown that obese insulin resistant subjects had fewer capillaries and a greater number of large vessels in WAT as compared to lean individuals.^89^ Together, these findings are indicative of vascular rarefaction and decreased vascular remodelling in WAT in obese humans. Thus, the lower capillary density may reflect higher WAT oxygenation in obesity. Alternatively, if WAT oxygenation would be lower in obesity, the pro‐angiogenic response is not effectively propagated.^90^

In addition to a lower capillary density in WAT of obese individuals, an increased vascular tone may impair ATBF, which ultimately determines tissue oxygen delivery. It is well established that ATBF is impaired in human obesity. Fasting ATBF is lower in obese compared to lean individuals and has been linked to insulin resistance.^59^, ^91^, ^92^, ^93^, ^94^, ^95^ Furthermore, in the postprandial period as well as during insulin stimulation (ie, hyperinsulinemic‐euglycemic clamp), the increase in ATBF is blunted in obese vs lean subjects.^59^, ^95^, ^96^ These impairments seem to be related to impaired beta‐adrenergic responsiveness and increased activity of the renin‐angiotensin system in obesity.^68^, ^94^, ^97^, ^98^ We have previously shown that both pharmacological and physiological manipulation of ATBF induced concomitant alterations in WAT pO_2_ in humans,^59^ suggesting that decreased ATBF in obesity indeed reduces AT oxygen supply. Importantly, however, WAT pO_2_ is not only determined by oxygen supply to the tissue but is also dependent on WAT oxygen consumption, as discussed in more detail below.

#### 3.1.2 Adipose tissue oxygen consumption and mitochondrial function

In normal weight individuals, WAT oxygen consumption is relatively low as compared to other tissues, accounting for approximately 5% of whole‐body oxygen consumption.^34^, ^62^, ^99^ It has been estimated that mitochondrial oxygen consumption accounts for up to 85%, while non‐mitochondrial oxygen consumption may be responsible for 10%‐15% of total oxygen consumption in WAT under steady‐state conditions.^100^, ^101^ Both mitochondrial and non‐mitochondrial oxygen consumption may change during the marked WAT remodelling occurring in obesity and may induce alterations in WAT oxygenation.

It is well established that mitochondrial morphology, mass and function are impaired in multiple adipose tissue depots in obese rodents.^102^, ^103^, ^104^, ^105^, ^106^ Interestingly, it has been reported that early in the development of obesity, enhanced mitochondrial metabolism, biogenesis and reactive oxygen species (ROS) production seem critical to initiate and promote adipocyte differentiation.^107^, ^108^ In line with findings in animals, several human studies have reported impaired mitochondrial capacity and reduced expression of genes/proteins related to mitochondrial metabolism (eg, peroxisome proliferator‐activated receptor gamma coactivator 1‐alpha and nuclear respiratory factor 1) in WAT in states of obesity, insulin resistance and T2DM.^34^, ^59^, ^109^, ^110^, ^111^, ^112^ Furthermore, it has been shown that mitochondrial proteins are downregulated not only at whole WAT level, but also in adipocytes from obese individuals.^113^, ^114^ In line, mitochondrial density and oxygen consumption rates are lower in adipocytes derived from obese vs lean subjects, independent of adipocyte size.^113^, ^114^, ^115^ Of note, there also appear to be sWAT depot‐specific differences in oxygen consumption rates in obesity, since basal respiration was lower in abdominal as compared to femoral differentiated human multipotent adipose‐derived stem cells.^116^ The latter finding may underlie the higher AT pO_2_ in abdominal than femoral subcutaneous adipose tissue.^116^

In accordance with impaired mitochondrial density and oxygen consumption in obese WAT in humans, there are indications that weight loss may evoke beneficial changes in WAT mitochondrial function. Following bariatric surgery, both mitochondrial respiratory capacity and biogenesis were increased in WAT.^117^, ^118^ We have recently shown that diet‐induced weight loss increased WAT gene expression of mitochondrial biogenesis markers and non‐mitochondrial oxygen consumption pathways in humans, which may have contributed to the reduction in WAT pO_2_ following weight loss.^74^ In contrast, instead of improving WAT mitochondrial abnormalities, weight loss downregulated mitochondrial gene expression and density, and had neither effects on mitochondrial DNA transcripts nor OXPHOS proteins.^119^ Interestingly, the latter study showed that a higher initial mitochondrial number and gene expression was related to more successful weight loss after 12‐month follow‐up. Importantly, however, changes in gene expression do not necessarily translate into functional alterations. Taken together, it appears that oxygen consumption is impaired in obese WAT in humans, which may contribute to increased WAT pO_2_ in human obesity.

## 4 ALTERED ADIPOSE TISSUE OXYGENATION MAY CONTRIBUTE TO TISSUE DYSFUNCTION AND METABOLIC IMPAIRMENTS

In cell culture experiments investigating the molecular and cellular responses to hypoxia, cells are usually exposed to a substantially reduced level of oxygen (1% O_2_ is frequently employed) as compared to “normoxia” (ambient air, 21% O_2_). The normal physiological range of AT pO_2_ in human WAT is ~3%‐11% O_2_ or ~23‐84 mmHg.^57^, ^59^, ^60^, ^73^ Therefore, the outcomes of experiments comparing the effects of pO_2_ below and well‐above these physiological levels should be interpreted with caution, since results may not directly translate to the human in vivo situation. Moreover, it is important to distinguish between acute (<24 h) and more prolonged exposure to different pO_2_ levels, since this seems to have a major impact on the metabolic and inflammatory responses, as will be discussed later in this section.

### 4.1 The cellular response to low oxygen levels

**Figure 2:**
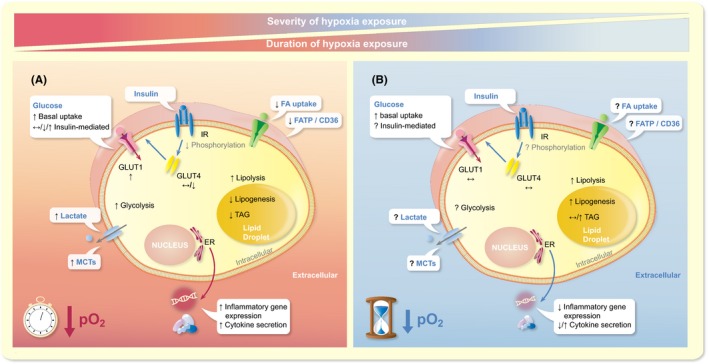
Adipocyte substrate metabolism, adipocyte gene expression and adipokine secretion are affected by alteration of oxygen partial pressure (pO_2_). Both the severity and the duration of hypoxia exposure seem to impact cellular processes, as explained in more detail in the text. Panel A shows the effects of acute exposure to severe hypoxia (usually 1% O_2_ for <24 h), while panel B illustrates the putative effects of prolonged, mild hypoxia exposure (usually 5%‐10% O_2_ for 7‐14 d) on adipocyte biology. ER, endoplasmic reticulum; FA, fatty acids; FATP/CD36, fatty acid transporters; GLUT, glucose transporter; IR, insulin receptor; MCTs, monocarboxylate transporters; pO_2_, oxygen partial pressure; TAG, triacylglycerol. ↑, increase; ↓, decrease; ↔, unchanged; ?, not determined

As any other cell type, adipocytes must maintain and adjust their metabolic and physiological regulation in response to fluctuations in the local microenvironment, including variation in oxygen levels.^25^, ^120^ The main regulators of oxygen sensing are the oxygen sensitive HIFs. HIFs are transcription factors, binding to the DNA and changing gene expression in response to alterations in oxygen levels.^121^ HIFs consist of two subunits, α and β, with the former being the oxygen sensitive molecule and HIF‐1β being constitutively expressed by cells.^67^ The HIF family consists of three members based on the three α‐subunits, HIF‐1α, HIF‐2α and HIF‐3α, with the predominant members being HIF‐1α and HIF‐2α.^27^, ^120^, ^122^ HIF‐1α has received the most attention, and this transcription factor has been described as the master regulator of oxygen homeostasis. HIF‐1α is continuously synthesized and rapidly degraded in the presence of oxygen but is stabilized when oxygen levels are low, and the functional HIF‐1α transcription factor is then recruited. More specific, during sufficient oxygenation of the cells, HIF‐1α is enzymatically degraded by prolyl‐4‐hydroxylases through the proteasome.^121^ During “hypoxic” conditions, which are tissue‐dependent, but usually defined as <1% of oxygen in most in vitro studies, the prolyl hydroxylase domain enzymes are inactivated, and HIF‐1α is not subject to rapid degradation. Instead, HIF‐1α then forms a heterodimer with the β subunit, acting on DNA binding areas called hypoxia‐responsive elements, thus regulating gene expression of many different genes.^10^, ^22^, ^25^, ^121^, ^123^ These genes encode proteins involved in a multiplicity of cellular processes, including glucose and lipid metabolism, inflammation, ECM metabolism and apoptosis.^25^ Thus, changes in tissue oxygenation seem to affect many physiological processes in WAT, and the metabolic and inflammatory effects will be discussed in more detail below (Figure 2).

### 4.2 Metabolic effects of altered adipose tissue oxygenation

#### 4.2.1 Glucose metabolism

Under hypoxic conditions, a shift from aerobic to anaerobic metabolism occurs, with glucose becoming the major substrate for ATP generation.^25^, ^67^, ^68^, ^121^ In vitro studies have demonstrated an increase in basal glucose uptake in human and rodent adipocytes treated acutely, up to 24 hours, with 1% vs 21% O_2_.^65^, ^124^, ^125^ Furthermore, it has been shown that glucose uptake in human adipocytes is inversely related to O_2 _levels (1, 3, 5, 10, 15% vs 21% O_2_), peaking at 1% O_2_.^126^ In accordance with these findings, prolonged exposure (14 d) to low (5% O_2_) but not high (10% O_2_) physiological pO_2_ levels tended to increase basal glucose uptake in differentiated human multipotent adipose‐derived stem cells.^116^

Conflicting findings, however, have been reported regarding the effects of pO_2_ on insulin‐mediated glucose uptake. Acute exposure to 1% O_2_ (up to 24 h) reduced insulin‐mediated glucose uptake in human adipocytes,^125^ indicative of impaired insulin signalling, an effect that was reversible.^125^ This was further illustrated by decreased phosphorylation of the insulin receptor, IRβ and IRS‐1 proteins as well as protein kinase B.^65^, ^125^ In contrast, another study found that acute 1% O_2_ exposure increased insulin‐dependent and insulin‐independent glucose uptake in 3T3‐L1 adipocytes.^127^ Interestingly, it was shown that multiple exposures of differentiating 3T3‐L1 adipocytes to transient hypoxia (1% O_2_, 4 h/d, 4‐8 d) enhanced insulin signalling, illustrated by increased phosphorylation of Akt (T308 and S473 residues) and GSK3β.^127^

Alterations in glucose uptake are because of changes in the expression and localization of the glucose transporters (GLUTs). GLUT‐1 mRNA levels were increased following exposure to acute, severe hypoxia (1%‐2% pO_2_, up to 24 h) in both murine (3T3‐L1) and human (pre)adipocytes.^67^, ^126^, ^128^, ^129^, ^130^, ^131^, ^132^, ^133^ In contrast, insulin‐dependent GLUT‐4 mRNA expression in human adipocytes remained unchanged 124 or was significantly reduced by acute exposure to 1% O_2_.^124^, ^126^, ^129^, ^132^ In line with improved insulin‐stimulated glucose uptake, GLUT‐4 but not GLUT‐1 expression was elevated in murine adipocytes exposed to transient hypoxia.^127^ During and after differentiation of human preadipocytes under low (5% O_2_) and high (10% O_2_) physiological pO_2_ levels, basal GLUT‐1 expression was not changed 134 or decreased,^116^ while GLUT‐4 mRNA expression remained unchanged.^116^, ^134^

Acute hypoxia exposure to 1% O_2 _for 24 hours also increased gene and protein expression of enzymes involved in glycolytic metabolism in human adipocytes, including glucose phosphate isomerase, pyruvate kinase and 6‐phosphofructo‐2‐kinase/fructose‐2,6‐biphosphatase.^128^, ^133^, ^135^, ^136^, ^137^ In accordance with these findings, the end‐product of the glycolytic pathway, lactate and the expression of genes encoding monocarboxylate transporters (MCT) mediating lactate transport were found to be increased in rodent and human adipocytes under hypoxic conditions.^67^, ^138^, ^139^

In conclusion, in vitro findings indicate that exposure to severe hypoxia (1%‐2% O_2_), and likely also low physiological pO_2_ (5% O_2_), increases basal glucose uptake and induces a switch towards glycolytic metabolism in rodent and human adipocytes, while effects on insulin‐mediated glucose uptake are conflicting (Figure 2).

#### 4.2.2 Lipid metabolism

Few studies examined whether and how pO_2_ influences lipid metabolism in WAT, yielding conflicting results. FFA uptake and oxidation were significantly reduced by acute, severe hypoxia exposure (1% O_2_, 24 h) in 3T3‐L1 adipocytes.^65^, ^127^ Reduced uptake may be explained by reduced expression of fatty acid transport proteins, as illustrated by decreased expression of FATP and CD36 in these cells.^65^ Lipid storage, assessed by TAG accumulation, was reduced both by chemically induced hypoxia with CoCl_2_ and prolonged severe hypoxia exposure in 3T3‐L1 adipocytes (1% O_2 _for 14 d).^140^, ^141^ In accordance with these observations, 1% O_2_ exposure for 14 days decreased lipogenesis in 3T3‐L1 adipocytes.^140^, ^141^ However, 14 days of exposure to mild hypoxia exposure (4% O_2_), which reflects low physiological pO_2_, markedly increased lipogenesis and the formation of large lipid droplets in 3T3‐L1 adipocytes.^140^ Furthermore, another study has shown that exposure of differentiating human adipocytes to high (10% O_2_) but not low (5% O_2_) physiological pO_2_ for 14 days increased TAG accumulation.^134^ Taken together, it seems that exposure of adipocytes to severe hypoxia may reduce lipogenesis, while prolonged exposure to physiological pO_2_ may increase lipogenesis, but these effects need to be studied in more detail to better understand the opposing results (Figure 2).

The amount of oxygen in the microenvironment also seems to impact adipocyte lipolysis. Several studies have shown that acute exposure to severe hypoxia (1% O_2_) increased basal lipolysis in 3T3‐L1 adipocytes.^65^, ^68^, ^125^ Moreover, prolonged exposure (14 d) to severe hypoxia modestly increased basal lipolysis, while low physiological pO_2_ (4% O_2_, 14 d) exposure increased lipolysis to a much greater extent in 3T3‐L1 adipocytes_._ 140 In theory, insulin resistance in adipocytes might explain the increased basal lipolytic rate because of reduced insulin‐mediated suppression of lipolysis. However, since improved insulin sensitivity has also been found following hypoxia exposure, as discussed in the previous section, alternative mechanisms are likely involved in the pO_2_‐induced effects on basal adipocyte lipolysis. Furthermore, isoproterenol‐induced lipolysis was also significantly elevated in human adipocytes differentiated at high (10% O_2_) and low (5% O_2_) physiological pO_2_ as compared to exposure to ambient air (21% O_2_), which was accompanied by increased protein expression of the lipolytic enzyme HSL and the lipid droplet‐coating protein perilipin.^134^ In conclusion, hypoxia seems to increase lipolysis in rodent and human adipocytes, with more pronounced effects found under physiological pO_2_ (Figure 2). Clearly, more studies are required before strong conclusions can be drawn regarding the effects of oxygenation on lipid metabolism in human WAT, and to unravel underlying mechanisms.

### 4.3 Adipokines and inflammatory factors

Several studies have demonstrated that the expression and secretion of many adipokines are sensitive to pO_2_ levels. Most in vitro studies have shown that acute exposure to severe hypoxia (1% O_2_, up to 24 h) induces a pro‐inflammatory expression and secretion profile in (pre)adipocytes, with increased levels of TNF‐α, IL‐1, IL‐6, monocyte chemoattractant protein‐1 (MCP‐1), plasminogen activator inhibitor (PAI)‐1, macrophage‐migration‐inhibition factor and inducible‐nitric oxide synthase, in both adipocytes and SVF cells derived from human adipose tissue, as well as in murine adipose tissue resident macrophages.^25^, ^41^, ^64^, ^142^ Furthermore, several studies found that acute exposure to severe hypoxia decreased adiponectin and increased leptin expression and secretion in human and murine (pre)adipocytes.^25^, ^63^, ^64^, ^65^, ^66^, ^129^, ^132^, ^133^, ^143^ Adiponectin, which is often reduced in individuals with obesity, is an important adipokine that has beneficial metabolic and anti‐atherogenic properties.^144^, ^145^ Leptin, the concentrations of which are strongly positively correlated to adipose tissue mass, is an important regulator of food intake and energy expenditure, providing important feedback in relation to energy storage in the body.^146^

As with other in vitro studies applying acute and severe hypoxia over 1‐24 hours,^25^, ^64^, ^66^, ^129^, ^132^, ^133^ these findings should be interpreted with some caution, underlining the importance of applying more physiological conditions in cell culture experiments. Few in vitro studies have tried to better mimic physiological conditions in vivo in terms of oxygen partial pressure as well as the duration of exposure to altered pO_2_.^116^, ^134^ The effects of modest, rather than severe, hypoxia have also been investigated, showing a concentration‐dependent change in adipokine expression and secretion in human adipocytes.^126^ Interestingly, prolonged exposure of human adipose tissue‐derived mesenchymal stem cells to physiological pO_2_ levels (ie, 5% and 10% O_2_) during differentiation towards mature adipocytes appears to elicit a different expression and secretion profile as observed following acute (severe) exposure to hypoxia. More specific, we have recently demonstrated that low physiological pO_2_ decreased pro‐inflammatory gene expression (ie, IL‐6, PAI‐I, TNFα, MCP‐1 and dipeptidyl‐peptidase‐4 [DPP‐4]) in differentiated human adipocytes as compared to 21% and/or 10% O_2_, whereas more heterogeneous effects on adipokine secretion were found.^116^ Exposure of these cells to low physiological pO_2_ (5% O_2_) for 14 days resulted in a reduced secretion of leptin and increased adiponectin and IL‐6 secretion in these adipocytes, while no significant effects on DPP‐4 and MCP‐1 secretion were found.^116^ In contrast, exposure to high physiological pO_2_ (10% O_2_) increased leptin and DPP‐4, but reduced IL‐6 and MCP‐1 secretion.^116^ Famulla and colleagues 134 have shown increased DPP‐4, adiponectin and IL‐6 following prolonged exposure to high physiological pO_2_ (10% O_2_), while low physiological pO_2_ (5% O_2_) tended to reduce the secretion of adiponectin. These differences between studies suggest that donor characteristics may also influence the effects of pO_2_ on the adipocyte secretory profile.

Taken together, oxygen levels and pattern of exposure seem to have a significant impact on adipocytokine expression and secretion (Figure 2). However, many aspects of exposure have not been examined in human cells, which is important to elucidate in future experiments.

## 5 ALTERED TISSUE OXYGENATION IMPACTS WHOLE‐BODY PHYSIOLOGY IN HUMANS

As indicated in the previous section, the cellular response to altered oxygen levels seems to depend to a large extent on the severity and duration of exposure. Not surprisingly, the effects of changes in oxygenation on whole‐body homeostasis also seems to be determined by these factors, next to the oxygenation pattern.^147^ The clinical consequences of severe chronic hypoxia, as observed in patients with severe chronic obstructive pulmonary disease (COPD), and severe intermittent hypoxia as seen in patients with obstructive sleep apnoea syndrome (OSAS) are outside the scope of this review and have been discussed elsewhere.^148^, ^149^, ^150^, ^151^, ^152^ In this section, we will provide a brief overview of findings on the effects of altered (adipose) tissue oxygenation through physiological or experimental conditions on body weight and parameters related to cardiometabolic health.

Living at high‐altitude represents a condition of hypobaric hypoxic exposure (ie, around 15% O_2_ at ~3000 m) as oxygen partial pressure is relatively lower compared to sea level.^147^ The impact of high‐altitude habitation on chronic diseases is dependent on several factors such as ethnicity, environmental and behavioural factors that may vary across mountain dwellers.^147^, ^153^ It has been suggested that living at high‐altitude is associated with improved cardiovascular and pulmonary function.^154^ Many studies have demonstrated a lower prevalence of obesity, cardiovascular diseases, T2DM and cancer in populations living at high altitude.^147^, ^153^, ^155^, ^156^, ^157^ For example, a cross‐sectional study including 422,603 adults has shown an inverse relationship between elevation and obesity prevalence, after adjusting for temperature, diet, physical activity, smoking and demographic factors, in both males and females,^158^ which is in line with other studies demonstrating an inverse association between altitude and the prevalence of obesity.^159^, ^160^, ^161^ Interestingly, a lower prevalence of the metabolic syndrome, lower reduced fasting glucose levels and diabetes incidence have been found among highlanders.^156^, ^162^, ^163^, ^164^, ^165^ Noteworthy, from most of these observational studies, it cannot be concluded that exposure to lower pO_2_ levels has beneficial health effects, since many potential confounders such as the diet and physical activity level may have affected these findings.

**Figure 3:**
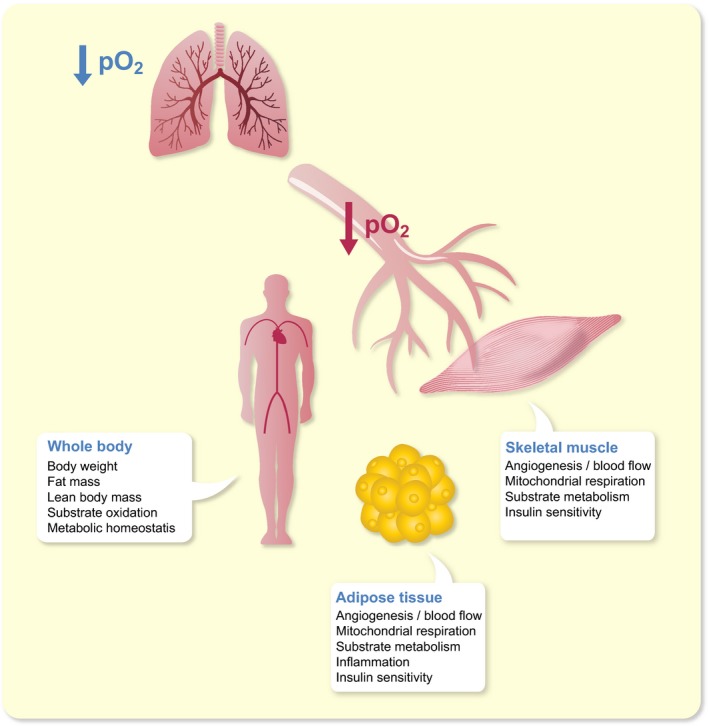
Putative impact of (moderate) hypoxia exposure on whole‐body, skeletal muscle and adipose tissue physiology. O_2_, oxygen; pO_2_, oxygen partial pressure

Several intervention studies have been performed to elucidate the impact of exposure to altered pO_2_ on body weight and metabolic homeostasis (Figure 3). We have previously demonstrated that chronic exposure to hypoxia (8% vs 21% O_2_, 21 d) improved the WAT phenotype in C57Bl/6J mice, evidenced by decreased adipocyte size, decreased macrophage infiltration and inflammatory markers and increased expression of mitochondrial function and biogenesis markers in visceral and subcutaneous AT.^166^ More recently, the same concept has been applied to humans. Exposure to moderate hypoxia (15% O_2_) for 10 subsequent nights increased whole‐body insulin sensitivity in eight obese men.^167^ Since moderate hypoxia exposure also tended to reduce AT pO_2_ 167, these findings may imply that lowering of AT pO_2_ by moderate hypoxia exposure may have contributed to improved insulin sensitivity.^168^ Furthermore, exposure to hypoxia under resting conditions increased energy expenditure and lipid metabolism, and reduced appetite and food intake.^169^, ^170^ Based on a recent systematic review, it was concluded that normobaric hypoxic conditioning, lasting from 5 days up to 8 months, may have beneficial effects on insulin levels, energy expenditure, body weight and blood pressure in rodents and humans, which may contribute to improved cardiometabolic health and body weight management in obesity.^155^ The putative effects of (severe) hypoxia exposure on orexigenic (ie, ghrelin) and anorexigenic (ie, leptin) peptides affecting appetite and food intake may, at least partially, underlie the effects on body weight and metabolic outcomes, as reviewed elsewhere.^171^, ^172^

Interestingly, the combination of hypoxia exposure and exercise may have additive beneficial health effects in humans.^147^, ^173^ A greater decrease in total body weight, body fat mass and waist/hip ratio was found when exercise was performed under hypoxia compared to normoxia,^170^, ^174^, ^175^, ^176^ and appeared to be maintained following the intervention.^177^ Interestingly, hypoxia exposure also seems to exert effects on substrate oxidation but findings are conflicting, with some studies showing increased fat oxidation,^178^, ^179^ while others demonstrating increased carbohydrate oxidation both during and post‐exercise.^180^, ^181^ Furthermore, exercise training under hypoxic conditions induced a more pronounced increase in adiponectin levels compared to normoxic exercise.^182^ Moreover, hypoxic exercise decreased insulin levels in obese individuals, and acutely improved insulin sensitivity in T2DM patients compared to normoxic exercise.^175^, ^183^, ^184^ The mechanisms underlying improvements in glucose homeostasis following hypoxia exposure remain to be elucidated, but may involve insulin‐independent mechanisms. Importantly, the impact of hypoxia on cardiometabolic health may also be because of effects of altered pO_2_ on other organs than adipose tissue, especially during exercise.

The beneficial effects of hypoxic exercise may be mediated to a large extent by alterations at the level of skeletal muscle. During contraction, glucose uptake in skeletal muscle is increased in an insulin‐independent manner, likely involving independent effects of 5' AMP‐activated protein kinase **(**AMPK), mechanical stress and Ca^2+^/calmodulin‐dependent protein kinase kinases (CaMKKs).^185^ Interestingly, it has been demonstrated that hypoxia exposure increased glucose uptake in skeletal muscle cells through AMPK signalling. Therefore, hypoxia exposure during exercise might have additive or synergistic effects on peripheral glucose uptake. Indeed, exposing human myotubes to 7% O_2_ in combination with electrical pulse stimulation (EPS), to mimic exercise, increased glucose uptake to a higher extent than EPS under 21% O_2_, which seems at least partly because of an insulin‐sensitizing effect of hypoxia.^186^ Taken together, hypoxia exposure may improve glucose homeostasis via insulin‐dependent and insulin‐independent effects, but more studies in humans on putative underlying mechanisms are needed.

## 6 CONCLUSIONS AND FUTURE PERSPECTIVES

The obesity epidemic presents a major public health challenge. Novel preventive measures and treatment alternatives are urgently needed to combat obesity and its complications. Adipose tissue dysfunction in obesity is related to a plethora of metabolic and endocrine disturbances, contributing to impairments in lipid and glucose metabolism as well as immune homeostasis. It is well established that adipose tissue dysfunction has a central role in the aetiology of obesity‐related comorbidities and chronic diseases, including T2DM and cardiovascular diseases. A reduced lipid buffering capacity of hypertrophic adipose tissue in obesity results in lipid accumulation in key metabolic organs such as the liver and skeletal muscle (ie, ectopic fat storage), which is strongly associated with insulin resistance. Moreover, adipose tissue in obesity is characterized by a pro‐inflammatory phenotype. This is reflected by a phenotypic shift towards a higher abundance of pro‐inflammatory macrophages and other adaptive and innate immune cells in obese adipose tissue, leading to the production and secretion of a multitude of pro‐inflammatory cytokines, which in turn may induce insulin resistance. Besides inflammation, a disproportionate deposition of ECM components during the development of obesity may contribute to adipose tissue fibrosis and insulin resistance (Figure 1).

Adipose tissue oxygen partial pressure, determined by the balance between oxygen supply and consumption, may have a key role in the metabolic and inflammatory perturbations seen in most obese individuals. Animal models have shown lower pO_2_ in obese WAT (“hypoxia”). Findings in humans are conflicting, which may be because of differences between study populations in terms of the onset and physical history (eg, weight cycling) of obesity and other subjects’ characteristics (eg, age, sex, ethnicity, presence of type 2 diabetes), the WAT depot studied, and the methodology used. Nevertheless, several studies performed in our laboratory indicate that AT pO_2_ is higher in obese insulin resistant individuals, is positively related to insulin resistance (independently of adiposity), and is reduced after diet‐induced weight loss, which is paralleled by improved insulin sensitivity. Adipose tissue mitochondrial dysfunction (ie, reduced O_2_ consumption) may contribute to higher AT pO_2_ in obesity. There is no strong evidence to suggest that differences in pO_2_ within the human physiological range (ie, because of impaired blood flow) have marked effects on mitochondrial respiration. Interestingly, many in vitro experiments have demonstrated that changes in oxygen levels impact the functionality of (pre)adipocytes and immune cells, leading to alterations in glucose and lipid metabolism, as well as inflammation in adipose tissue (Figure 2). Clearly, altered pO_2_ may not only affect adipose tissue physiology but also whole‐body metabolic homeostasis (Figure 3). In this respect, it remains to be elucidated whether AT pO_2_ exerts a crucial role in the development and progression of obesity‐related complications in humans. Although several lines of evidence suggest that exposure to lower levels of oxygen may enhance whole‐body metabolic homeostasis and body weight regulation, intervention studies in humans are warranted to further investigate whether changes in tissue oxygenation may improve cardiometabolic health, thereby providing a novel strategy to combat chronic cardiometabolic diseases in obese humans.

## CONFLICT OF INTEREST

The authors have declared that no conflict of interest exists.
